# Syrup of Tar

**Published:** 1889-11

**Authors:** 


SYRUP OF TAR.—The prevalence of influenza throughout an extensive region makes anything in the line of cough mixtures desirable, and we give the following method of preparing syrup of tar, from *N. E. Med. Monthly*.—Mr. F. W. Haussmann says, in the *Pharmaceutical Era*, June, 1889, that the following method of making syrup of tar is the least troublesome of all, requires less time, and is the most cleanly:

| | |
|---|---|
| Oil of tar, dark | f ℥ ss. |
| Magnesia, carbonated, | ℥ ss. |
| Sugar, granulated, | 14 ozs. |
| Hot water, | a sufficient quantity. |

Rub the oil with the magnesia and two ounces of the sugar, and add gradually eight ounces of the water. Allow the mixture to stand twelve hours and filter. In the filtrate dissolve the rest of the sugar without heat.

This method furnishes a permanent, dark-brown preparation of strong tarry taste.

